# A comprehensive study of deaths due to exposure to humidifier disinfectant in Korea: focusing on medical records, assessment of exposure to humidifier disinfectants, and causes of death

**DOI:** 10.4178/epih.e2021091

**Published:** 2021-11-01

**Authors:** Yeong Jun Ju, Seungho Lee, Seungsoo Sheen, Dong-Woo Choi, Jong-Han Leem, Soon Young Lee

**Affiliations:** 1 Department of Preventive Medicine and Public Health, Ajou University School of Medicine, Suwon, Korea; 2 Department of Occupational and Environmental Medicine, Ajou University School of Medicine, Suwon, Korea; 3 Department of Pulmonary and Critical Care Medicine, Ajou University School of Medicine, Suwon, Korea; 4 Data Link and Operation Team, Cancer Data Center, National Cancer Control Institute, National Cancer Center, Goyang, Korea; 5 Department of Occupational and Environmental Medicine, Inha University Hospital, Incheon, Korea; 6 Department of Social and Preventive Medicine, Inha University School of Medicine, Incheon, Korea

**Keywords:** Humidifiers, Disinfectants, Cause of death, Medical records, Deceased victims

## Abstract

**OBJECTIVES:**

We aimed to determine the characteristics of the deceased victims of deaths caused by exposure to humidifier disinfectants, and present the distribution of the victims’ data submitted for damage application, demographic characteristics, imaging findings, characteristics of humidifier disinfectant exposure, and distribution of the causes of death.

**METHODS:**

An integrated database of victims was established using the medical records data of 1,413 victims submitted during the application for death damage caused by exposure to humidifier disinfectants, and the demographic characteristics, medical records, imaging findings, exposure characteristics, and cause of death were examined.

**RESULTS:**

The average numbers of data submissions of each applicant for death damage were 3.0 medical use records. A total of 608 (43.0%) victims had more than one finding of acute, subacute, or chronic interstitial lung diseases. The average daily and cumulative use times of the victims were 14.40 and 24,645.81 hours, respectively, indicating greater exposure in this group than in the survivors. The humidifier disinfectants’ components comprised polyhexamethylene guanidine (72.8%), chloromethylisothiazolinone/methylisothiazolinone (10.5%), other components (15.0%), and oligo-[2-(2-ethoxy)-ethoxyethyl] guanidine chloride (1.5%). The components’ distribution was 67.8% for single-component use, which was higher than that in the survivors (59.8%). The distribution of the causes of death were: respiratory diseases (54.4%), neoplasms (16.8%), and circulatory diseases (6.3%). Other interstitial lung diseases (65.5%) were the most common cause of death among those who died due to respiratory diseases.

**CONCLUSIONS:**

Careful discussions of appropriate remedies should be conducted based on a comprehensive understanding of the characteristics of the deceased victims, considering their specificities and limitations.

## INTRODUCTION

The humidifier disinfectant accident was an unprecedented major environmental disaster that occurred in Korea due to the use of household goods [1]. Humidifier disinfectants containing chemicals, oligo-[2-(2-ethoxy)-ethoxyethyl] guanidine chloride (PGH), polyhexamethylene guanidine (PHMG), and chloromethylisothiazolinone/methylisothiazolinone (CMIT/MIT), as their main components, were first launched in Korea in 1994. Approximately 40 types of humidifier disinfectants were sold on the market in 2011, which were estimated to have been used by approximately 8 million people [2].

Various health-related problems caused by the exposure to humidifier disinfectants have been reported, including physical diseases, psychiatric problems, and pulmonary diseases, and damage cases are still being reported [3-6]. According to a previous epidemiological study investigating the actual conditions of the applicants for damage caused by exposure to humidifier disinfectants, worsening or diagnosis of diseases after exposure to humidifier disinfectants in adult victims (survivors) was reported: pulmonary diseases (83.0%), nasal diseases such as rhinitis (71.0%), skin diseases such as dermatitis (56.6%), ophthalmic diseases such as conjunctivitis (47.1%), gastritis/gastric ulcer (46.7%), and cardiovascular diseases (42.2%). Moreover, insomnia (55.9%), depression (50.8%), anxiety disorder (39.6%), and post-traumatic stress disorder (39.1%) worsened after exposure to humidifier disinfectants [7]. In other words, these study results suggest that the health problems caused by exposure to humidifier disinfectants are not limited to pulmonary diseases, but can be accompanied by various physical and psychiatric problems. As of July 2, 2021, a total of 7,490 victims had applied for damage support (5,813 survivors and 1,677 deceased victims). Of the total applicants, only 54.3% (n=3,159) and 60.7% (n=1,018) were recognized as survivors and deceased victims, respectively, and received financial assistance for damage caused by this exposure; moreover, a significant number of applicants for damage support remain unrecognized [8].

If the objective data for damage determination, such as medical records data and radiological findings, are submitted, the damage recognition can be discussed in detail; however, if the relevant data are not submitted, a passive approach is inevitable due to the absence of objective data, and several factors may limit the recognition of damage. In particular, the deceased victims are the most prone to these limitations, with many difficulties remedying the damage because they have already died and cannot submit data to prove their health damage. Considering these specificities and limitations of victims who died of health damage due to exposure to humidifier disinfectants, the damage remedy for them should be comprehensively discussed; therefore, a basic understanding of the characteristics of victims who died due to exposure to humidifier disinfectants should be considered.

Thus, this study aimed to report the characteristics of individuals who had applied for death damage caused by the exposure to humidifier disinfectants for the first time in Korea, to present the distribution of the data submitted by these applicants, their demographic characteristics, imaging findings, characteristics of exposure to humidifier disinfectants, and distribution characteristics of the causes of death.

## MATERIALS AND METHODS

### Data source and measurements

#### Medical records data

The medical records data of 1,413 applicants for death damage (as of December 31, 2019) were provided by the National Institute of Environmental Research (NIER). A database was established by reviewing all submitted medical records of each individual and by classifying them using the deceased victims’ personal identification codes, medical use information, test results (chest X-ray, chest computed tomography [CT], pulmonary function test, biopsy, and cytology), and information on exposure to humidifier disinfectants. Two health information managers constructed the database and performed data cleansing under the supervision of a pulmonologist under cross-coding according to the variable frame, and the data were finally completed and reviewed by a statistical expert after the revision. For the X-ray or CT findings, data were coded after a comprehensive review by several pulmonologists.

#### Investigation and determination of the data on health damage caused by exposure to humidifier disinfectants

Health damage caused by exposure to humidifier disinfectant is defined as “any damage to life or health caused by exposure to a humidifier disinfectant containing toxic chemical substances” under the Enforcement Decree of the Special Act on Remedy for Damage Caused by Humidifier Disinfectants, and those who wish to be recognized as having experienced health damage caused by exposure to humidifier disinfectants have to apply to the Comprehensive Support Center for Victims of Humidifier Disinfectants of the Korea Environmental Industry and Technology Institute (KEITI). The procedures in recognizing health damage caused by exposure to humidifier disinfectants is as follows: (1) When a victim applies. (2-1) Environmental exposure investigation (investigation of the use environment, period of use, and product used as a humidifier disinfectant) and (2-2) medical investigation (clinical, imaging, and histopathological investigations based on the medical record documents submitted by the applicant) are conducted. (3) Based on these findings, the special committee for examination performs a comprehensive determination of the health damage. (4) The committee on remedying the damage caused by exposure to humidifier disinfectants deliberates and resolves the final examination and determination results. Lastly, (5) the committee informs the applicant of the final outcome.

After the revision of the Special Act on September 25, 2020, the scope of the recognition was expanded to comprehensively recognize damage and determine the grades of injury by reviewing the overall health condition without specifying the disease; the distinction between remedial benefits and remedy accounts was abolished, and the criteria for both categories were integrated into the remedial benefits. The information on the health damage examination and determination of humidifier disinfectants used in this study were provided by the KEITI, with data on the damage determination of 1,413 people who had submitted damage applications in December 31, 2019. Recognition information on health damage due to pulmonary disease, fetal damage, asthma, adult interstitial lung disease (ILD), pediatric ILD, bronchiectasis, and pneumonia damage are included. Considering the current status of support for damage caused by exposure to humidifier disinfectants, which is not limited to specific diseases but broadens the scope of damage recognition in this study, the victims’ health damage was recognized and diagnosed as due to any of the following diseases: stage 1 or stage 2 of pulmonary disease during remedial benefit determination, fetal damage, asthma, or stage 3 pulmonary disease during remedial account determination, adult/child ILD, bronchiectasis, pneumonia, or asthma. If health damage caused by at least one of the above mentioned diseases was recognized, the individual was classified as a defined victim. If it was not recognized in the damage recognition examination and determination, the individual was classified as a unrecognized victim.

#### Environmental exposure data

The environmental exposure investigation involved data analysis of deaths using the data of applicants for damage caused by exposure to humidifier disinfectants, obtained from the KEITI. The investigation was performed as follows. An experienced investigator confirmed the applicant’s personal information by phone and schedules a visit. Next, a face-to-face investigation was conducted when visiting the place where the humidifier disinfectant was used (residence or workplace). The exposure investigation table prepared while conducting the investigation was reconfirmed and constructed by coding the contents on the table as environmental exposure investigation data. The investigation contents include the use of and status of exposure to humidifier disinfectants, including the time of disinfectant use, the period from the start to the end of the exposure to the disinfectant, total months of use, the distance between the humidifier and the respiratory organ, the direction of humidifier spraying, the use of humidifier disinfectants when sleeping, the location of use, and the amount of onetime use (unit: cc). Details in this regard can be found in the previous study [9].

#### National Health Insurance Service data

This study used the data of a customized sample cohort from 2002 to 2018 obtained from the National Health Insurance Service (NHIS). Using identifiable information of the deceased victim received from the NIER, data related to the victims’ deaths were extracted from the Microdata Integrated Service of Statistics Korea and linked to customized sample cohort data to determine the cause of death.

Since the NHIS’s customized sample cohort data were only accessible until December 31, 2018, the causes of death of 1,341 people were identified, excluding those of 72 individuals who had died since January 1, 2019.

### Statistical analysis

This study analyzed the distribution of data submitted by the applicants for death damage, their demographic characteristics, imaging findings, and characteristics of exposure to humidifier disinfectants by linking the medical record data of applicants for death damage caused by exposure to humidifier disinfectant, investigation and determination data of health damage caused by exposure to humidifier disinfectant, and environmental exposure data. Additionally, the distribution characteristics of the causes of death among applicants were analyzed using the NHIS customized sample cohort. The specific analyses performed were as follows: First, the distribution of the data submitted by the applicants for death damage was identified by analyzing the database constructed using the medical record data of death damage applicants. Second, to analyze the difference in characteristics depending on whether or not health damage caused by the exposure to humidifier disinfectant is recognized, the general characteristics and imaging findings of the applicants for death damage were analyzed by recognition of health damage. Third, the distribution of the daily humidifier disinfectant use times, cumulative exposure time, and components used in the humidifier disinfectant were evaluated by analyzing the environmental exposure data of the applicants for death damage. Lastly, the distribution of the causes of death of the damage applicants was evaluated by analyzing the NHIS customized cohort data, and the distribution was analyzed by classifying the total causes of death and deaths caused by respiratory system diseases (International Statistical Classification of Diseases [ICD]: J00-J99). All data processing and statistical analyses were performed using SAS version 9.4 (SAS Institute Inc., Cary, NC, USA).

### Ethics statement

This study was approved by the Institutional Review Board of Ajou University Hospital (IRB No. AJIRB-20-047).

## RESULTS

### Distribution of data submitted by applicants for death damage

Supplementary Material 1 shows the distribution of the damage application data submitted by 1,404 people, excluding nine who did not submit their medical records data among 1,413 applicants for death damage. The average numbers of data submissions of each applicant were 3.0 for medical use records, 25.3 for X-ray results, 3.5 for CT results, 4.8 for pulmonary function test results, 1.3 for biopsy results, and 4.1 for cytology results, with the X-ray results being the highest.

### General characteristics of each health damage recognition caused by exposure to humidifier disinfectants

The general characteristics of 1,413 applicants for death damage are presented in Table 1. Of them, 755 were male (53.4%) and 649 were female (45.9%); nine applicants did not submit their medical records data. In terms of age, the proportion of adults aged 19-64 years (33.9%) and those aged 65 years or older (33.6%) was high, while the proportion of infants and children (0-1 year: 8.3%; 2-6 years: 7.0%) was relatively higher than that of adolescents. Regarding past medical history, hypertension, malignant neoplasms, diabetes, and tuberculosis were the most common among the defined victims caused by exposure to humidifier disinfectants. Meanwhile, those who were unrecognized had malignant neoplasms, hypertension, diabetes, and tuberculosis, in order. Particularly, victims with a history of malignant neoplasms were more likely to be unrecognized damage caused by humidifier disinfectants (recognized: 43.9%, unrecognized: 56.1%).

### Characteristics of imaging findings depending on whether health damage caused by exposure to humidifier disinfectants is recognized or not

Table 2 presents the distribution of findings in the recognized and unrecognized groups by classifying interstitial lung diseases into acute, subacute, and chronic. A total of 68 individuals (6.5%) were diagnosed with acute interstitial pneumonia (AIP), while 127 (12.2%) were diagnosed with bronchiolitis obliterans organizing pneumonia (BOOP) and cryptogenic organizing pneumonia (COP). Non-specific interstitial pneumonia (NSIP) and idiopathic pulmonary fibrosis were diagnosed in 221 (21.2%) and 431 individuals (41.3%), respectively. Moreover, 64 individuals (6.1%) were diagnosed with hypersensitivity pneumonitis. Based on the submitted multiple medical records data, 608 (43.0%) individuals had more than one case of relevant CT findings and more than one findings of interstitial lung disease. Among them, 496 individuals were recognized, while 112 were unrecognized. Overall, differences were observed between the recognized and unrecognized groups. One or more diagnostic findings regarding to the lung diseases (AIP, BOOP/COP, and NSIP) were detected in the recognized group, while the corresponding findings were rarely observed in the unrecognized group.

### Exposure characteristics of the deceased

**Figure 1.**
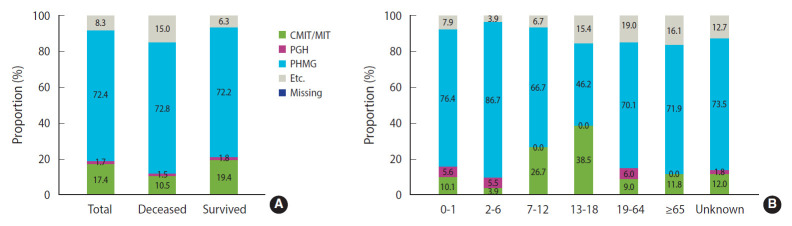
Overall distribution of the components used in humidifier disinfectants (A) and distribution by age (B). CMIT/MIT, chloromethylisothiazolinone/methylisothiazolinone; PGH, oligo-[2-(2-ethoxy)-ethoxyethyl] guanidine chloride; PHMG, polyhexamethylene guanidine.

**Figure 2.**
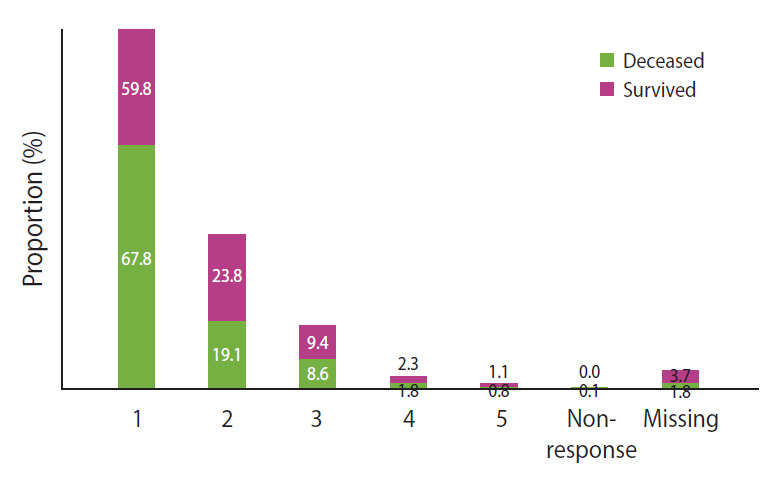
Distribution of the number of humidifier disinfectants used.

The average daily humidifier disinfectant use times were 14.40 hours for deceased victims and 12.93 hours for survivors (Table 3). The total cumulative exposure times to humidifier disinfectants were 24,645.81 hours in the deceased victims and 20,888.22 hours in the survivors (Table 4). The distribution of each component of the humidifier disinfectant used by the deceased victims of deaths due to exposure to humidifier disinfectants and the results presented by age are shown in Figure 1. Regarding the components of the humidifier disinfectants used by the deceased victims, PHMG was the most common component (72.8%), followed by other components (15.0%), CMIT/MIT (10.5%), and PGH (1.5%), and CMIT/MIT (19.4%) among survivors, whose usage was higher than that among deceased victims. When the components used were compared by age, the distribution was generally similar, but the rate of CMIT/MIT use was higher in children and adolescents than in other age groups. The number of disinfectants used by applicants for damage caused by exposure to humidifier disinfectants is presented in Figure 2. Among the applicants for death damage, 67.8% used only one humidifier disinfectant, while 32.2% used a combination of more than two disinfectants. Among the surviving applicants for damage, 59.8% used only one humidifier disinfectant, while 40.2% used a combination of more than two disinfectants, which was a higher percentage of duplicate use compared with that of the applicants for death damage.

### Distribution of the causes of death

**Figure 3.**
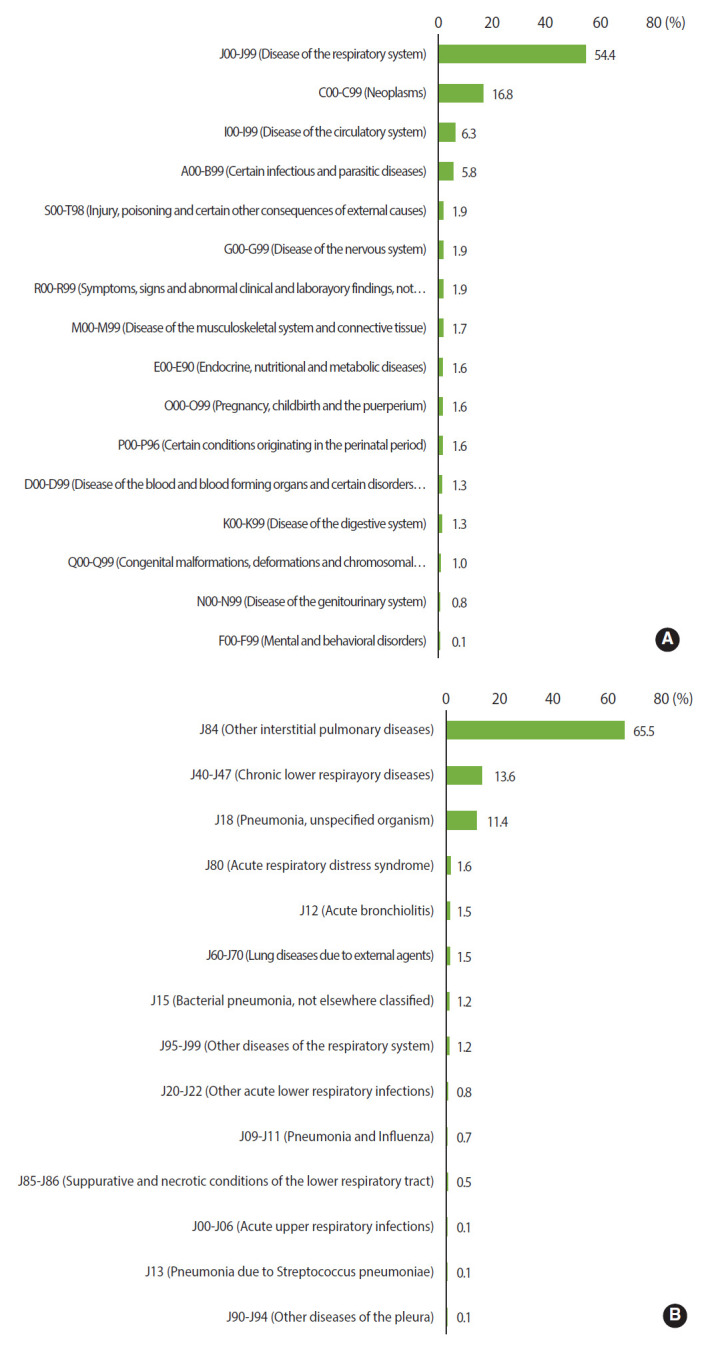
Distribution of all causes of death (A) and distribution of causes of death due to respiratory diseases (J00-J99) (B).

The distribution of the causes of death for 1,341 deceased victims is presented in Figure 3. The most common causes of death were respiratory system diseases (J00-J99; n=730, 54.4%), neoplasms (C00-C99; n=225, 16.8%), and circulatory system diseases (I00-I99; n=84, 6.3%). Among the respiratory system diseases, other interstitial lung diseases (J84) was the common, and followed by chronic lower respiratory tract diseases (J40-J47), and pneumonia due to unspecified organisms (J18). Those were accounted for 478 (65.5%), 99 (13.6%), and 83 (11.4%), respectively.

## DISCUSSION

This study reported the characteristics of deceased victims caused by exposure to humidifier disinfectants using their medical records, investigation and determination data of health damage caused by humidifier disinfectants, environmental exposure data, and NHIS data. Considering the limitations and specificities of the remedy for death damage caused by humidifier disinfectants, the characteristics of deceased victims were comprehensively presented so that the remedy for damage could be comprehensively discussed.

This study, which included 1,413 applicants for death damage caused by exposure to humidifier disinfectants, observed similar demographic characteristics with those of 4,482 victims who survived from exposure to humidifier disinfectants included in a previous study [9]. Males were more predominant in this study than females. With regard to the age characteristics, the distribution of children aged 0-12 years among the deceased victims was relatively noteworthy (17.2%), which was similar to that of children aged < 10 years in the study of survivors from exposure to humidifier disinfectants (15.5%).

The characteristics of exposure to humidifier disinfectants of the deceased victim were compared with those of the survivors. To our knowledge, this was the first study to report the exposure characteristics of deceased victims caused by humidifier disinfectants in Korea, and it was impossible to compare them with those of previous studies. Hence, the exposure investigation data of applicants for damage caused by exposure to humidifier disinfectants owned by the research team were used and compared with those of the survivors. The average daily use time (mean± standard deviation) was 14.40±6.74 hours for the deceased victims, which was longer than that of the surviving victims (12.93± 5.93 hours). Similarly, the cumulative exposure time to humidifier disinfectants was 24,645.81± 37,960.78 hours for deceased victims, which was longer than that for the surviving victims (20,888.22± 30,113.05 hours). Although the absolute exposure amount cannot be compared, it can be inferred from the study results that the deceased victims were relatively more exposed to humidifier disinfectants than the survivors.

The characteristics of the causes of death of the victims of humidifier disinfectant exposure were compared with the statistical distribution of the causes of death in the Korean population in 2018 [10]. Regarding the distribution of the causes of death among deceased victims who had applied for death damage caused by the exposure to humidifier disinfectants, respiratory system-related deaths had the highest proportion: respiratory system disease (54.4%), malignant neoplasms (16.8%), and circulatory system disease (6.3%); conversely, the distribution of the causes of death in the Korean population differed: malignant neoplasms (27.0%), circulatory system disease (21.1%), and respiratory system disease (12.6%). When subdividing the respiratory system diseases, other interstitial lung diseases were the most common among the deceased victims (65.5%), followed by chronic lower respiratory tract diseases (13.6%), and pneumonia due to unspecified organisms (11.4%). In comparison, the distribution of the causes of death in the Korean population was slightly different: pneumonia (61.6%), other chronic obstructive pulmonary diseases (12.4%), and other diseases of the respiratory system (10.0%). These results prove that the damage and death caused by respiratory system diseases, which rarely occur in the general population, were sufficiently severe to lead to death in the group affected by humidifier disinfectants.

Applications for damage due to humidifier disinfectants have continued from the first application in November 2011 to the fifth application in current. With the revision of the Special Act on Remedy for Damage Caused by Humidifier Disinfectants in September 2020, there have been specific changes to the wide recognition of damage by reviewing the overall health status. However, 40% of the deceased victims among the applicants for damage caused by humidifier disinfectants are still not recognized, leading to blocking of the remedy for damage. In the case of the deceased victims, they have already died and can no longer submit data to prove their health damage. In particular, infants and children are likely to have insufficient evidence such as imaging data and clinical progress notes due to sudden death. Moreover, it is complicated for non-professionals to recognize infrequent and unique health damage, such as specific health damage caused by humidifier disinfectants, to prepare related data for damage recognition and prove the health damage. Hence, a comprehensive discussion is warranted to ensure appropriate remedy for the damage by considering the characteristics of the applicants for death damage along with the data submitted by them. Based on the results of this study, the applicant characteristics that can be considered in the process of comprehensive discussion are as follows. First, it is necessary to attain a comprehensive determination by linking the medical record data with the NHIS data in consideration of the fact that the number of submitted medical records is relatively low, with an average of 3.0 cases per individual, and that it is impossible to submit data in the future. Second, since applications for many infants and children were submitted for death damage caused by exposure to humidifier disinfectants, who may have unique characteristics compared with the general population, discussion with various expert groups, including those from related fields, may be necessary to clarify the characteristics of this patient population.

In summary, many applicants for death damage caused by humidifier disinfectants have not been recognized, and the complexity and various issues of damage determination are piling up. Hence, further efforts should be made to appropriately remedy the applicants for damage and continue the conduct of related studies and discussions [11,12]. In particular, the specific characteristics of the applicants for death damage caused by humidifier disinfectants and limitations of remedy for damage should be considered. The appropriate remedy should be provided through comprehensive discussions involving various expert groups to understand the detailed characteristics of applicants for death damage.

This study is significant because it is the first study to report the characteristics of deceased victims of deaths caused by exposure to humidifier disinfectants in Korea. However, since it does not affect the determination results of the victims, this study focused on the causes of death and exposure characteristics of the deceased victims, excluding the part of the determination results as much as possible. The limitations of this study are as follows. First, the causes of death in the statistics database provided by Statistics Korea could not identify the precise cause of death because the ICD code used was not subdivided. Second, regarding the past medical history, since the analysis was based on the data submitted by the applicant when receiving damage, data on patient’s disease history that were not submitted could not be considered. Lastly, if the characteristics of deaths from damage caused by humidifier disinfectants were compared with those of the general population group or the group that did not use humidifier disinfectants, more meaningful conclusions, such as differences in characteristics between the humidifier disinfectant victim group and the general group, could be drawn; but a comparison could not be made due to the lack of relevant studies.

## Figures and Tables

**Table 1.** Deceased victims’ general characteristics

Characteristics		Total (n=1,413)	Damage caused by humidifier disinfectant			
			Recognized (n=911)^1^		Unrecognized (n=502)^2^	
		n (%)	n (%)	Row percent (%)	n (%)	Row percent (%)
Sex						
	Male	755 (53.4)	492 (54.0)	65.2	263 (52.4)	34.8
	Female	649 (45.9)	414 (45.4)	63.8	235 (46.8)	36.2
	Unknown	9 (0.6)	5 (0.5)	55.6	4 (0.8)	44.4
Age (yr)						
	0-1	117 (8.3)	81 (8.9)	69.2	36 (7.2)	30.8
	2-6	99 (7.0)	82 (9.0)	82.8	17 (3.4)	17.2
	7-12	17 (1.2)	10 (1.1)	58.8	7 (1.4)	41.2
	13-18	10 (0.7)	8 (0.9)	80.0	2 (0.4)	20.0
	19-64	479 (33.9)	309 (33.9)	64.5	170 (33.9)	35.5
	≥65	475 (33.6)	284 (31.2)	59.8	191 (38.0)	40.2
	Unknown	216 (15.3)	137 (15.0)	63.4	79 (15.7)	36.6
Past history						
	Hypertension	341 (24.1)	210 (23.1)	61.6	131 (26.1)	38.4
	Diabetes	211 (14.9)	141 (15.5)	66.8	70 (13.9)	33.2
	Thyroid disease	39 (2.8)	27 (3.0)	69.2	12 (2.4)	30.8
	Hepatitis	51 (3.6)	29 (3.2)	56.9	22 (4.4)	43.1
	Tuberculosis	199 (14.1)	137 (15.0)	68.8	62 (12.4)	31.2
	COPD	101 (7.1)	60 (6.6)	59.4	41 (8.2)	40.6
	Cancer	237 (16.8)	104 (11.4)	43.9	133 (26.5)	56.1
	Asthma	90 (6.4)	58 (6.4)	64.4	32 (6.4)	35.6
	Lung disease	109 (7.7)	75 (8.3)	68.8	34 (6.8)	31.2
	Bronchiectasis	27 (1.9)	20 (2.2)	74.1	7 (1.4)	25.9
	Pneumonia	53 (3.8)	41 (4.5)	77.4	12 (2.4)	22.6

COPD, chronic obstructive lung disease; HDLI, humidifier disinfectant-associated lung injury; ILD, interstitial lung disease.

1 Where injury was recognized as caused by one or more of the following diseases: HDLI (definite, probable, or possible), fetal injury, asthma, adult’s ILD, children’s ILD, bronchiectasis, and pneumonia.

2 All the rest.

**Table 2.** CT findings for interstitial lung disease according to damage caused by humidifier disinfectants

CT findings	Total	Damage caused by humidifier disinfectant	
		Recognized^1^	Unrecognized^2^
AIP	68 (6.5)	65 (8.9)	3 (1.0)
BOOP/COP	127 (12.2)	107 (14.6)	20 (6.4)
NSIP	221 (21.2)	181 (24.7)	40 (12.9)
UIP/IPF	431 (41.3)	341 (46.5)	90 (28.9)
HP	64 (6.1)	52 (7.1)	12 (3.9)
Missing	369 (28.8)	178 (19.3)	191 (53.7)

Values are presented as number (%). CT, computed tomography; AIP, acute interstitial pneumonia; BOOP, bronchiolitis obliterans organizing pneumonia; COP, cryptogenic organizing pneumonia; NSIP, non-specific interstitial pneumonia; UIP, usual interstitial pneumonia; IPF, idiopathic pulmonary fibrosis; HP, hypersensitivity pneumonitis; HDLI, humidifier disinfectant-associated lung injury; ILD, interstitial lung disease.

1 Where injury was recognized caused by one or more of the following diseases:HDLI (definite, probable, or possible), fetal injury, asthma, adult’s ILD, children’s ILD, bronchiectasis, and pneumonia.

2 All the rest.

**Table 3.** Average usage time (hour) per day of humidifier disinfectants

Category	Frequency	Min	Max	Median	Mode	Mean±SD
Deceased	1,311	1.0	24.0	12.0	24.0	14.40±6.74
Survived	4,724	0.5	24.0	11.0	24.0	12.93±5.93

Min, minimum; Max, maximum; SD, standard deviation.

**Table 4.** Distribution of cumulative exposure time (hour) to humidifier disinfectants^1^

Category	Frequency	Min	Max	Median	Mode	Mean±SD
Deceased	1,311	12.0	360,864.0	11,760.0	4,032.0	24,645.81±37,960.78
Survived	4,724	12.0	400,512.0	10,920.0	6,720.0	20,888.22±30,113.05

Min, minimum; Max, maximum; SD, standard deviation.

1 Cumulative exposure time was calculated by multiplying period of use (month) by monthly average usage weeks (week), average usage day by week (day), and average daily usage time (hour).

## SUPPLEMENTARY MATERIALS

Supplementary Material 1. Characteristics of the medical records of deceased victims

Korean version is available at https://www.e-epih.org/.
