# Supplementary material for: A comprehensive study of deaths due to exposure to humidifier disinfectant in Korea: focusing on medical records, assessment of exposure to humidifier disinfectants, and causes of death
Source: Epidemiol Health. 2021 Nov 1;43:e2021091. doi: 10.4178/epih.e2021091 (PMC8920737; doi:10.4178/epih.e2021091)
Supplement: Supplementary Material 1. — Characteristics of the medical records of deceased victims [file epih-43-e2021091-suppl1.docx]

Supplementary Material 1. Characteristics of the medical records of deceased victims

| **Category** | **Total** | **Average** | **S.D** | **Median** | **Max** |
| --- | --- | --- | --- | --- | --- |
| Medial use record | 1,404 | 3.0 | 3.15 | 2 | 27 |
| X-ray | 1,120 | 25.3 | 31.8 | 15 | 252 |
| CT | 1,046 | 3.5 | 3.78 | 2 | 60 |
| Pulmonary function test | 600 | 4.8 | 6.55 | 2 | 67 |
| Biopsy | 377 | 1.3 | 0.70 | 1 | 6 |
| Cytology | 348 | 4.1 | 7.24 | 2 | 100 |
