# Supplementary material for: A comprehensive study of deaths due to exposure to humidifier disinfectant in Korea: focusing on medical records, assessment of exposure to humidifier disinfectants, and causes of death
Source: Epidemiol Health. 2021 Nov 1;43:e2021091. doi: 10.4178/epih.e2021091 (PMC8920737; doi:10.4178/epih.e2021091)
Supplement: Supplementary file 2 [file epih-43-e2021091-suppl2.docx]

**가습기살균제 사망 피해자들의 특성 연구:
의무기록, 가습기살균제 노출평가, 사망원인을 중심으로**

**A comprehensive study of deaths due to exposure to humidifier disinfectant: Focusing on medical records, assessment of exposure to humidifier disinfectants, and causes of death**

**Running title:** A comprehensive study of deaths due to humidifier disinfectants

Yeong Jun Ju, PhD ^1,^ **^†^**, Seungho Lee, PhD ^2,^ **^†^**, Seungsoo Sheen, MD, MS ^3^,
Dong-Woo Choi, PhD ^4^, Jong-Han Leem, MD, PhD ^5,6^, Soon Young Lee, MD, PhD ^1, *^

^1^Department of Preventive Medicine and Public Health, Ajou University School of Medicine, Suwon, Korea

^2^Department of Occupational and Environmental Medicine, Ajou University School of Medicine, Suwon, Korea

^3^Department of Pulmonary and Critical Care Medicine, Ajou University School of Medicine, Suwon, Korea

^4^Data Link & Operation Team, Cancer Data Center, National Cancer Control Institute, National Cancer Center, Goyang, Korea

^5^Department of Occupational and Environmental Medicine, Inha University Hospital, Incheon, Korea

^6^Department of Social and Preventive Medicine, Inha University School of Medicine, Incheon, Korea

^†^ Denotes equal contribution

***Corresponding author:** **Soon Young Lee**

Department of Preventive Medicine and Public Health, Ajou University School of Medicine 206 World Cup-ro, Yeongtong-gu, Suwon-si Gyeonggi-do 16499, Republic of Korea

**E-mail**: solee@ajou.ac.kr

**초록**

**연구목표:** 이 연구는 가습기살균제 피해 신청자들 중 사망자의 특성에 대한 기본근거 산출을 목표로 하며, 사망 피해 신청자의 피해신청 제출자료 분포, 인구학적 특성, 영상소견 특성, 가습기살균제 노출 특성, 사망원인 분포 특성을 제시하고자 한다.

**연구방법:** 가습기 살균제 사망 피해 신청자 1,413명을 대상으로, 피해신청시 제출된 의무기록자료를 이용하여 사망자 통합 데이터베이스를 구축하였으며, 사망자들의 인구학적 분포, 의료이용기록, 영상소견 등을 살펴보았다. 또한 가습기 살균제 노출자료를 이용하여 가습기살균제 노출 시간, 살균제 노출성분을 분석하였으며, 국민건강보험공단자료와 통계청 사망원인자료를 연계하여 사망 피해 신청자들의 주요 사망원인 분포를 확인하였다.

**연구결과:** 사망 피해 신청자의 피해 신청 제출자료 개인별 평균 제출건수는 의료이용기록 3.0건, X-ray 25.3건, CT 3.5건, 폐기능검사 4.8건, 조직검사 1.3건, 세포학검사 4.1건으로 X-ray의 평균 제출건수가 가장 많았다. 일반적 특성을 살펴보면, 전체 사망피해자들의 연령별 구성은 0~1세 8.3%, 2~6세 7.0%, 7~12세 1.2%, 13~18세 0.7%, 19~64세 33.9%, 65세 이상 33.6% 이었다. 급성, 아급성, 만성 간질성 폐질환 소견이 한 건 이상 있는 대상자는 608명으로 전체 43%에 해당한다. 사망 피해 신청자의 가습기살균제 노출 특성의 경우 사망피해자들의 일일평균 사용 시간과 평균누적사용시간은 각각 12.9시간, 24,645시간으로 생존자보다 노출이 시간이 많았다. 가습기살균제 사용 성분의 경우에는 PHMG (72.8%), CMIT/MIT (10.5%), 기타 (15.0%), PGH (1.5%)순으로 생존 신청자 보다 CMIT/MIT 사용 분율이 낮은 반면 기타 성분의 사용 분율이 높은 특성이 있었다. 가습기살균제 사용 성분 분포는 사망 피해 신청자에서 단일성분 단독사용 분율이 67.8%로 생존 피해 신청자의 59.8% 보다 높았다. 사망원인분포의 경우 호흡계통질환 (54.4%), 신생물 (16.8%), 순환계통질환 (6.3%) 순이었다. 호흡계통질환 사망자의 사망원인을 세부적으로 보면 기타 간질성 폐질환이 65.5%로 가장 많았다.

**결론:** 가습기살균제 사망 피해자 집단이 가지는 특수성과 제한점을 고려하여 이들에 대한 포괄적인 특성이해를 바탕으로 적정 구제를 위한 세심한 논의를 이루어 가야 함을 제안한다.

**키워드:** 사망원인, 노출특성, 가습기살균제 피해자, 사망

**연구배경**

가습기살균제 사고는 한국에서 발생한 세계적으로 유례없는 생활용품에 의한 대규모의 환경참사이다 [1]. 화학물질 PGH (oligo (2-(2ethoxy)ethoxyethyl guanidine), PHMG (polyhexamethylene guanidine), CMIT/MIT (chloromethyl-isothiazolione/methyl-isothiazolinone)를 주요 성분으로 하는 가습기살균제는 1994년 국내 첫 출시를 시작으로 2011년까지 대략 40여 종의 가습기살균제가 시판되었으며, 약 800만명이 사용한 것으로 추정된다 [2].

가습기살균제 사용으로 인한 건강피해는 다양하게 보고 되었는데 특히 폐질환 뿐 아니라 다양한 신체질환과 정신과적 문제를 동반하여 나타나고 있으며 현재까지도 피해사례가 접수되고 있다 [3-6]. 가습기살균제 피해 신청자의 실태를 조사한 역학연구에 따르면 성인피해자(생존자)에서 가습기살균제 노출 이후 악화 또는 진단받은 질환은 폐질환 (83.0%), 비염 등 비질환 (71.0%), 피부염 등 피부질환 (56.6%), 결막염 등 안과질환 (47.1%), 위염·궤양(46.7%), 심혈관계질환(42.2%) 순으로 보고되었다. 또한 가습기살균제 노출 이후 불면증 (55.9%), 우울증 (50.8%), 불안장애 (39.6%), 외상 후 스트레스장애 (39.1%) 가 악화되었다는 응답이 보고되었다 [7]. 이와 같은 연구결과들을 종합해보면 가습기살균제 노출로 인한 건강문제가 폐질환에 국한되지 않고 다양한 신체질환과 정신과적 문제를 동반 할 수 있음을 시사한다. 2021년 7월 2일 기준으로 가습기살균제 피해를 신청한 접수자는 총 7,490명으로 생존자 5,813명과 사망자 1,677명의 피해신청이 접수되었다. 전체 신청자들 중 생존자에서는 54.3% (3,159명), 사망자에서는 60.7% (1,018명)만이 피해인정을 통해 피해지원을 받았으며, 아직 판정이 진행되고 있음을 고려하더라도 상당수의 피해신청자들이 여전히 피해를 인정받지 못하고 있는 실정이다 [8].

의무기록자료와 영상의학소견 등 피해판정을 위한 객관적인 자료들이 제출된 경우에는 피해인정에 대한 논의가 구체적으로 진행될 수 있는 반면, 관련 자료 제출이 이루어지지 않은 경우에는 객관적 자료 부재로 인해 소극적으로 접근할 수 밖에 없으며 피해인정에 대한 많은 제한점들을 수반하게 된다. 특히 이러한 제한점에 가장 많이 노출되어 있는 대상자들이 사망 피해 신청자들인데 이미 사망하여 더 이상 건강피해를 입증할 수 있는 자료를 제출 할 수 없어 피해구제의 어려움이 많다. 가습기 살균제 사망 피해 신청자의 이러한 특수성과 제한점을 고려하여 이들에 대한 피해 구제가 종합적으로 논의되어야 하며, 이를 위해서는 가습기살균제 사망 피해자들의 특성에 대한 기본적인 이해가 선행되어야 할 것이다.

이에 이 연구는 국내에서는 처음으로 가습기살균제 사망 피해 신청자들에 대한 특성 보고를 목표로 하여 사망 피해 신청자의 피해신청 제출자료 분포, 인구학적 특성, 영상소견 특성, 가습기살균제 노출 특성, 사망원인 분포 특성을 제시하고자 한다.

**연구방법**

**연구데이터**

1. 의무기록자료

국립환경과학원에서 전달받은 사망 피해 신청자 1413명(2019년 12월 31일 기준)의 의무기록자료를 제공받았으며 제출된 개인별 모든 의무기록 자료를 검토하여 사망자 개인식별코드, 의료이용정보, 검사결과 (흉부X선, 흉부CT, 폐기능검사, 조직검사, 세포학검사), 가습기살균제 노출정보로 분류하여 데이터베이스를 구축하였다. 데이터베이스 구축 및 자료 정제는 호흡기내과 전문의의 감독아래 보건의료정보관리사 2인이 변수 틀에 따라 교차코딩으로 진행하였고 수정작업 후 통계전문가의 검토를 통해 최종적으로 자료를 완성하였다. 특히, X-ray나 CT 판독소견의 경우 다수의 호흡기내과 전문의의 종합 검토를 거쳐 자료를 코딩하였다.

1. 가습기살균제 건강피해 조사 · 판정 자료

가습기살균제 건강피해는 가습기살균제 피해구제를 위한 특별법에 따라 ‘독성 화학물질을 함유한 가습기살균제에 노출됨으로써 발생한 생명 또는 건강상의 피해’ 로 정의되며 가습기살균제 건강피해를 인정받고자 하는 대상자가 한국환경산업기술원 가습기살균제종합지원센터로 신청하도록 되어있다. 가습기살균제 건강피해 인정절차는 다음과 같다. 1) 피해 신청자가 접수를 하면 2-1) 환경노출조사 (가습기살균제 사용환경, 사용기간 및 사용제품 등을 조사), 2-2) 의학적 조사 (신청자가 제출한 진료기록 서류를 바탕으로 임상, 영상, 조직병리 등을 조사)를 진행하며 3) 이를 바탕으로 조사 · 판정 전문위원회에서 건강피해 종합판정을 내리고 4) 가습기살균제 피해구제위원회에서 최종 조사 · 판정 결과를 심의 · 의결하여 5) 신청자에게 최종결과를 안내한다.

한편, 기존에는 폐질환, 태아피해, 천식피해, 아동 · 성인 간질성 폐질환, 기관지 확장증, 폐렴 등 특정질환을 중심으로 건강피해를 판단하였으며 이와 같은 특정질환으로 피해를 인정받게 되면 정부재정으로 지원하는 ‘구제급여’ 와 기업 자금으로 지원하는 ‘구제계정’을 통해 피해를 구제받을 수 있었다. 하지만, 2020년 9월 25일 특별법이 개정되면서 질환을 특정하지 않고 전체적인 건강 상태를 검토하여 포괄적인 피해인정, 피해등급을 판정하는 것으로 인정범위가 확대되었고, 구제급여와 특별구제계정을 구분하던 것을 폐지하고 구제급여로 통합하였다. 이 연구에서 사용된 가습기 살균제 건강피해 조사 · 판정 정보는 2019년 12월 31일 까지 피해 신청이 접수된 1,413명의 가습기살균제 피해판정 정보를 한국환경산업기술원으로부터 제공받았다. 각 피해 신청자의 폐질환, 태아피해, 천식, 성인 ILD, 소아 ILD, 기관지확장증, 폐렴 피해에 대한 건강피해 인정정보가 포함되어 있다. 특정질환에 국한하지 않고 피해 인정범위를 포괄적으로 넓히고 있는 현재의 가습기살균제 피해지원 현황을 고려하여, 이 연구에서는 건강피해 인정자의 경우 구제급여 판정 중 폐질환 1,2단계, 태아피해, 천식 또는 구제계정 판정 중 폐질환 3단계, 성인 · 소아 ILD, 기관지확장증, 폐렴, 천식 중 어느 질환으로 든 하나 이상 피해가 인정된 경우에 피해 인정으로 구분하였으며 피해인정 조사 · 판정 에서 불인정 된 경우 피해 불인정자로 분류하였다.

1. 환경노출자료

환경노출조사는 한국환경산업기술원에 가습기살균제로 인한 피해신청자를 대상으로 조사된 자료 중 사망자에 대한 자료를 제공받아 분석하였다. 간단하게 언급하면 다음과 같다. 숙련된 조사원이 전화로 피해 신청자의 인적사항을 확인하고 방문일정을 잡는다. 다음으로 가습기살균제를 사용했던 장소 - 거주지 혹은 직장에 방문하여 대면조사를 실시한다. 조사를 수행하며 작성한 노출조사표는 재확인 후 코딩작업을 통해 환경노출조사자료로 구축된다. 또한, 각 조사원은 피해자 별 조사보고서를 작성하여 보다 객관적인 판정을 할 수 있도록 한다. 조사 참여에 동의한 신청자들을 대상으로 가능한 피해신청자를 직접 조사하였으나 피해자가 사망하거나 아주 어린 경우는 대리인을 통해 당시 상황에 대한 구체적 조사를 진행하였다. 조사내용은 살균제 사용시간, 살균제 노출 시작 ~ 종료기간, 총 사용 개월, 가습기와 호흡기과의 거리, 가습기 분무방향, 취침 때 가습기살균제 사용 관련 내용, 사용장소, 일회 사용량 (cc) 등 가습기살균제 사용현황과 노출현황에 대해 조사하고 있다. 이와 관련하여 자세한 점은 선행연구에서 확인할 수 있다 [9].

1. 국민건강보험공단 자료

이 연구는 2002년부터 2018년까지의 국민건강보험공단 맞춤형 표본 코호트 데이터를 사용하였다. 국립환경과학원으로부터 전달받은 사망자 식별정보를 이용하여 통계청의 마이크로데이터 통합서비스에서 사망자의 사망정보를 추출하여 맞춤형 표본 코호트 데이터와 연계하여 사망자들의 사망원인을 확인하였다.

국민건강보험공단 맞춤형 표본 코호트 데이터 자료는 2018년 12월 31일까지의 자료만 접근 가능하여 2019년 1월 1일 이후 사망자 72명을 제외하고 총 1,341명의 사인을 확인하였다.

1. 통계분석

이 연구는 가습기 살균제 사망 피해신청자의 의무기록자료, 가습기살균제 건강피해 조사 · 판정 자료, 환경노출자료를 연계하여 사망 피해 신청자의 피해신청 제출자료 분포, 인구학적 특성, 영상소견 특성, 가습기살균제 노출 특성을 분석하였다. 또한 국민건강보험공단 맞춤형 표본 코호트를 통해 사망 피해 신청자의 사망원인 분포 특성을 분석하였다. 구체적인 분석 내용은 다음과 같다. 첫번째, 사망 피해 신청자의 의무기록자료로 구축된 데이터베이스 분석을 통해 사망피해 신청자의 제출자료 분포를 파악하였다. 두번째, 가습기살균제 건강피해 인정 여부 별 특성 차이를 분석하기 위해 사망 피해 신청자의 건강피해 인정 여부 별 일반적 특성과, 영상소견 특성을 분석하였다. 세번째, 사망 피해 신청자의 환경노출자료 분석을 통해 가습기살균제 일일 사용 시간, 누적노출시간, 가습기살균제 사용 성분의 분포를 분석하였다. 마지막으로, 국민건강보험공단 맞춤형 코호트 자료 분석을 통해 사망 피해 신청자의 사망원인 분포를 분석하였으며 전체 사망 원인과, 호흡기계통 질환 (한국표준질병 · 사인분류: J00-J99)에서의 사망원인을 분류하여 분포를 분석하였다. 모든 자료처리 및 통계분석은 SAS version 9.4 (SAS Institute, Cary, NC, USA)로 수행하였다.

6. 연구윤리

이 연구는 아주대학교병원 기관연구 윤리심의위원회의 연구윤리 심의를 받았다 (IRB No. AJIRB-20-047).

**연구결과**

**사망 피해 신청자의 제출자료 분포**

전체 1,413명의 사망 피해 신청자들 중 의무기록 자료를 제출하지 않은 9명을 제외한 1,404명이제출한 피해 신청 자료의 분포는 Table 1에 제시하였다. 개인별 평균 제출건수를 살펴보면 의료이용기록 3.0건, X-ray 25.3건, CT 3.5건, 폐기능검사 4.8건, 조직검사 1.3건 세포학검사 4.1건으로 X-ray의 평균 제출건수가 가장 많았다.

**가습기살균제 건강피해 인정 여부 별 일반적 특성**

사망 피해 신청자 1,413명의 일반적 특성은 Table 2에 제시하였다. 성별 분포는 남성 755명 (53.4%), 여성 649명 (45.9%), 의무기록 자료를 제출하지 않은 대상자 9명으로 구성되어 있었다. 연령별로 보면 성인 (19~64세: 33.9 %) 및 65세 이상 장년층(33.6 %)의 분율이 높았으며 영유아 (0~1세: 8.3 %, 2~6세: 7.0 %)의 분포가 청소년층에 비해 상대적으로 높았다. 과거력의 경우 가습기살균제로 인한 피해 인정자에서는 고혈압, 악성신생물, 당뇨병, 결핵 순이었으며, 불인정자에서는 악성신생물, 고혈압, 당뇨병, 결핵 순이었다. 특히, 악성신생물을 과거력으로 가지고 있는 피해자에서 가습기살균제로 인한 피해가 불인정 된 경우가 인정된 경우보다 많았다 (인정: 43.9%, 불인정: 56.1%).

**가습기살균제 건강피해 인정 여부 별 영상소견 특성**

Table 3에서 간질성 폐질환을 급성, 아급성, 만성으로 구분하여 피해인정군과 비인정군에서의 소견 분포를 제시하였다. 급성 간질폐렴 (Acute Interstitial Pneumonia, AIP) 소견이 있었던 케이스는 68건 (6.5%), 폐쇄세기관지 기질화 폐렴 (Bronchiolitis Obliterans Organizing Pneumonia, BOOP)과 특발성 기질화 폐렴 (Cryptogenic Organizing Pneumonia, COP) 소견이 있었던 케이스는 127건 (12.2%)이었다. 비특이성 간질성폐렴 (Non-Specific Interstitial Pneumonia, NSIP)과 특발성 폐섬유화증 (Idiopathic Pulmonary Fibrosis, IPF) 소견은 각각 221건 (21.2%), 431건 (41.3%)이었다. 이 밖에 과민성 폐렴 (Hypersensitivity Pneumonitis, HP) 소견은 64건 (6.1%) 이었다. 개인이 여러 건의 의무기록 자료를 제출한 경우 관련 CT 소견이 한 개 이상인 케이스가 존재하며 전체 간질성폐질환 소견이 한 건 이상 있는 대상자는 608명이었다. 이중 인정받은 케이스는 496건, 불인정 케이스는 112건이었다. 전체적으로 보면 인정자와 불인정자에서 차이가 있었는데 인정자에서는 급성 간질폐렴, 폐쇄세기관지 기질화 폐렴 또는 특발성 기질화 폐렴, 비특이성 간질성폐렴에 대한 소견이 상당수 관찰된데 반해 불인정자 에서는 인정자에 비해 해당소견들이 드물었다.

**사망자들의 노출특성**

가습기살균제 일 평균 사용시간은 사망자의 경우 14.4 시간이었고, 생존자의 경우 12.9시간으로 사망자에서 일 평균 사용시간이 더 높았다 (Table 4). 가습기살균제 총 누적노출시간은 사망자에서는 24,645시간, 생존자에서는 20,888시간으로 사망자에서 더 높았다 (Table 5). 가습기살균제 사망 피해자들이 사용한 가습기살균제 성분 별 분포와 이를 연령별로 제시한 결과는 Fig. 1에 제시되었다. 사망피해자들의 가습기살균제 사용 성분은 PHMG가 72.8%로 가장 많았으며 기타 15.0%, CMIT/MIT 10.5%, PGH 1.5%순이었으며, 생존자에서는 CMIT/MIT 성분 사용분율이 19.4%로 사망자보다 더 높았다. 연령별로 사용성분을 비교해보면 대체로 비슷한 분포를 보이나 어린이와 청소년층에서 CMIT/MIT 성분 사용분율이 다른 집단에 비해 높았다. 가습기살균제 피해 신청자들의 사용한 살균제의 개수는 Fig. 2에 제시되었다. 사망 피해 신청자 중 가습기살균제를 1개만 사용한 단독사용한 경우는 67.8%이며 2개이상 중복사용은 32.2%였다. 생존 피해 신청자에서는 단독사용 59.8%, 2개이상 중복사용 40.2%로 사망 피해 신청자에 비해 중복사용 분율이 더 높았다.

**사망원인 분포**

사망원인 정보가 존재하는 사망자 1341명의 원사인의 분포는 Fig. 3에 제시되었다. 사망원인으로 가장 많은 분포를 차지한 것은 호흡계통질환 (J00-J99; n=730명), 신생물 (C00-C99; n=225명), 순환계통질환 (I00-I99; n=84명) 순이었다. 전체의 54.4%에 해당하는 호흡계통질환 사망자의 사인을 세부적으로 보면 기타 간질성 폐질환 (J84)가 478명으로 65.5%에 해당하고, 만성 하기도질환 (J40-J47)이 99명 (13.6 %), 상세불명 병원체의 폐렴이 83명 (11.4 %)로 뒤를 이었다.

**연구고찰**

이 연구는 가습기살균제 사망 피해자의 의무기록자료, 가습기살균제 건강피해 조사 · 판정 자료, 환경노출자료, 국민건강보험공단 자료를 이용하여 가습기살균제 사망 피해자의 특성을 보고한 연구이다. 가습기 살균제 사망 피해 신청자의 피해구제의 제한점과 특수성을 고려하여 이들에 대한 피해 구제가 종합적으로 논의될 수 있도록 사망 피해자의 특성을 포괄적으로 제시하고자 하였다.

총 1,413명의 가습기살균제 사망 피해 신청자를 대상으로 한 이 연구는 가습기살균제 생존피해자 4,482명을 대상으로 한 연구와 비교했을 때 인구학적 특성면에서 비슷한 특성을 관찰할 수 있었다 [9]. 기존 생존피해자를 대상으로 한 선행연구와 마찬가지로 우리 연구에서도 연구대상자 중 남성이 여성보다 조금 더 높은 분포를 차지하였다. 연령특성으로 보면 사망피해자 중 0세 ~ 12세 까지의 아동의 분포가 17.2%로 비교적 주목 할 만한 분포였는데 생존피해자의 연구에서도 10세미만의 아동의 분포가 15.5%로 비슷하였다.

가습기살균제 사망피해자들의 가습기살균제 노출 특성을 생존피해자와 비교해 보면 다음과 같다. 저자들이 파악하기로는 현재 가습기살균제 사망피해자의 노출특성에 대한 보고가 국내에서는 처음 이루어 진 것으로 선행연구를 통한 비교가 불가하며, 생존피해자에 대해서도 비교할 수 있는 선행연구가 제한되어 연구진이 보유하고 있는 가습기살균제 피해 신청자의 노출조사 데이터를 활용하여 생존피해자와 비교하였다. 하루평균 사용시간의 경우 사망피해자는 14.40±6.74 (평균±표준편차) 시간으로 생존피해자의 12.93±5.93시간에 비해 하루평균 사용시간이 더 길었다. 가습기살균제 누적노출 시간도 마찬가지로 사망피해자에서는 24645.81±37960.78시간으로 생존피해자의 20888.22±30.113.05시간보다 누적노출 시간이 길었다. 절대적인 노출량을 비교할 수는 없지만 연구결과를 통해 가습기살균제 사망피해자는 생존피해자에 비해 상대적으로 가습기살균제에 더 많이 노출되었음을 짐작할 수 있다. 하지만 이결과를 통해 노출특성 (노출량, 노출시간 등)과 건강피해의 관계를 파악하기에는 큰 제한점이 있으며 이에 대해서는 추가 연구를 바탕으로 논의되어야 함을 제안한다. 가습기살균제 성분에 대해서는, 사망피해자에서는 PHMG사용 72.8%, 기타 (PHMG, CMIT/MIT, PGH 외)성분사용 15.0%, CMIT/MIT사용 10.5%, PGH사용 1.5% 였으며 생존피해자에서는 PHMG사용 72.2%, 기타 (PHMG, CMIT/MIT, PGH 외)성분사용 6.3%, CMIT/MIT사용 19.4%, PGH사용 1.8%로 비슷하였으나 CMIT/MIT사용에 있어서는 다소 차이가 있었다.

통계청 마이크로데이터 통합서비스에서 제공하는 원사인 사망원인 통계자료를 우리 연구대상자들과 식별하여 사망원인 특성을 도출하였다. 가습기살균제 사망피해자의 사망원인 특성을 가장 최근자료인 2018년 대한민국 인구의 사망원인 통계 분포와 비교해 보면 다음과 같다 [10]. 사망피해자에서의 사망원인 분포는 호흡계통 (54.4%), 악성 신생물 (16.8%), 순환계통 (6.3%) 순으로 호흡계통 사망원인이 큰 분포를 차지하는데 반해 대한민국 인구의 사망원인 분포는 악성 신생물 (27.0%), 순환계통 질환 (21.1%), 호흡계통 질환 (12.6%) 순으로 차이가 있었다. 호흡계통질환을 세분화해서 들여다보면, 사망피해자 에서는 기타간질성폐질환이 65.5%로 가장 많았으며 만성하기도질환 (13.6%), 상세불명병원체의폐렴 (11.4%) 순인데 반해 대한민국 인구의 사망원인 분포는 폐렴 (61.6%), 기타 만성 폐쇄성 폐질환 (12.4%), 나머지 호흡계통의 질환 (10.0%) 순으로 다소 차이가 있었다. 이러한 결과는 보통 인구집단에서는 비교적 드문 호흡계통으로 인한 피해와 사망이 가습기살균제로 인한 피해 집단에서는 사망에 이르게 할 만큼 그 피해가 극심했음을 방증한다.

가습기살균제로 인한 피해 신청은 2011년 11월 1차 신청부터 현재의 5차 신청까지 피해 신청이 지속되고 있다. 2020년 9월 가습기살균제 피해구제를 위한 특별법이 개정되면서 전체적인 건강 상태를 검토하여 포괄적인 피해 인정을 위한 구체적인 변화가 있었지만, 여전히 가습기살균제 피해 신청자들 중 사망자들의 약 40%가 건강피해를 인정받지 못해 피해구제가 막혀 있다. 사망자들의 경우 이미 사망하여 더 이상 건강피해를 입증할 수 있는 자료를 제출 할 수 없으며, 특히 영유아의 경우 급작스런 사망으로 영상자료, 임상경과 기록 등 증빙할 만한 자료가 충분하지 않을 가능성이 높다. 우리 연구결과에서 사망 피해 신청자들이 제출한 개인별 의료이용 기록은 3.0건이었는데, 가습기살균제로 인한 특정 건강피해처럼 매우 드물게 특수하게 진행되는 건강피해를 전문가가 아닌 일반인이 인지하여 피해 인정을 위해 관련 자료를 준비하고 건강피해를 입증해 내기는 매우 어렵다. 사망 피해 신청자들이 제출한 자료와 더불어 이들의 특성을 종합적으로 고려하여 적정 피해 구제가 이루어 지도록 종합적인 논의가 필요할 것이다. 우리 연구결과를 바탕으로 종합적인 논의의 과정에서 고려해 볼 수 있는 사망 피해 신청자들의 특성은 다음과 같다. 첫째, 제출된 의료이용 기록이 개인당 평균 3.0건으로 매우 적은 점과 앞으로 자료 제출이 불가한 점을 고려하여 의무기록 자료를 국민건강보험공단 자료와 연계하여 종합적으로 판단할 수 있도록 하는 것이 필요하다. 두 번째, 가습기살균제 사망 피해를 신청한 영유아 집단이 상당한데, 영유아 집단은 일반 인구 집단에 비해 특수한 임상특성을 가질 수 있으므로 관련 전문가를 포함하여 다양한 전문가 집단이 참여하는 논의가 필요하다.

종합해보면, 가습기 살균제 사망 피해 신청자 들 중 건강피해를 인정받지 못한 신청자들이 여전히 많이 남아있으며 피해판정의 복잡성과 다양한 이슈들이 산적해 있어 피해 신청자들의 적정 구제를 위해 앞으로 더 많은 노력을 기울어야 하는 현실을 인지하고 관련 연구와 논의를 끊임없이 이어 나가야 할 것이다 [11-12]. 특히, 가습기 살균제 사망 피해 신청자의 특수성과 피해구제의 제한점을 고려하여야 하며, 기계적인 건강피해 판정을 지양하고 사망 피해 신청자 중심의 세밀한 특성 파악을 바탕으로 다양한 전문가집단이 참여하는 종합적인 논의를 통해 적정구제를 이루어 나가야 함을 제안한다.

이 연구는 국내 가습기살균제 사망 피해자들의 특성에 대한 최초의 연구라는 점에 의의가 있다. 다만 피해자들의 판정결과에는 어떠한 영향을 미치지 않는 만큼 본 연구에서는 가능한 판정결과에 대한 부분을 배제하고 사망자들의 사인과 노출특성 등에 대해 초점을 맞추었다. 본 연구의 제한점은 다음과 같다. 첫째, 통계청에서 제공하는 사망원인통계 데이터베이스의 원사인은 사망원인질환에 대한 ICD 코드가 세분류되어 있지 않아 자세한 사망원인을 식별 할 수 없었다. 또한 자료이용의 제한 때문에 의무기록자료 및 노출특성자료들과 연계분석을 할 수 없어 사망 피해자들의 사인분포와 국내 2018년 전체 사인분포를 비교하였다. 두번째, 과거력의 경우 피해 접수시 신청자가 제출한 자료에 근거한 분석이므로 제출하지 않은 자료의 질병력을 고려할 수는 없다. 마지막으로, 가습기살균제 피해로 인한 사망자의 특성을 일반인구집단이나 가습기살균제를 사용하지 않은 집단과 비교하였으면 가습기살균제 피해자 집단과 일반집단에서의 특성 차이 등 더 의미있는 결론을 도출 할 수 있을 것이나 관련 연구가 존재하지 않아 비교를 할 수 없었다. 하지만, 가습기살균제 피해신청자 집단 중 생존자 집단과 비교, 사망자 집단 중 건강피해 인정집단과 불인정 집단의 비교를 통해 사망자 집단의 특성을 다양하게 비교하고자 노력하였다. 이와 관련해서는 우리 연구뿐만 아니라 가습기살균제 피해 관련 모든 연구들의 제한점이며 앞으로 더 많은 역학연구를 통해 가습기살균제 피해 집단에 대한 특성 파악과 이들 집단에 대한 적정구제 방안 등 필요한 논의 등이 시급히 이루어져야 함을 제안한다.

**Acknowledgement**

이 연구는 환경부의 재원으로 국립환경과학원의 지원을 받아 수행한 것으로 연구의 결과는 국립환경과학원과 관련이 없음을 밝힌다 (NIER-SP2020-145).

**그림 목차**

Fig. 1 가습기살균제 사용성분 전체 분포 (왼쪽), 나이별 분포 (오른쪽)

Fig. 2 사용한 가습기살균제 개수 분포

Fig. 3 모든 사망원인 분포 (왼쪽), 호흡계통질환 사망원인 분포 (J00-J99, 오른쪽)

**참고문헌**

1. KCDC. Interim report of epidemiologic investigation of lung injury with unknown causes in Korea (in Korean). Korea Center for Disease Control and Preventation 2011

2. Choi JE, Hong S-B, Do K-H, Kim HJ, Chung S, Lee E, et al. Humidifier disinfectant lung injury, how do we approach the issues? Environmental health and toxicology 2016;31

3. Yoo S, Sim M, Choi J, Jeon K, Shin J, Chung S, et al. Psychological responses among humidifier disinfectant disaster victims and their families. Journal of Korean medical science 2019;34

4. Leem JH, Joh J-S, Hong Y-S, Kim J, Park S, Lim S, et al. Characteristics of a new respiratory syndrome associated with the use of a humidifier disinfectant: humidifier disinfectant-related respiratory syndrome (HDRS). International Journal of Occupational Medicine and Environmental Health 2020;33:829-839.

5. Paek D, Koh Y, Park D-U, Cheong H-K, Do K-H, Lim C-M, et al. Nationwide study of humidifier disinfectant lung injury in South Korea, 1994–2011. Incidence and dose–response relationships. Annals of the American Thoracic Society 2015;12:1813-1821.

6. Leem JH, Chung KH. Combined approaches using adverse outcome pathways and big data to find potential diseases associated with humidifier disinfectant. Environmental health and toxicology 2017;32

7. Kim DH, Lee SY, Leem JH, Lee SH, Kim YH, Yoo MS, et al. Household survey for the victims due to humidifier disinfectants. Seoul: Special Investigation Commission on Humidifier Disinfectant Disaster and April 16 Sewol Ferry Disaster; 2020, p. 59-111 (Korean).

8. Korea Environmental Industry & Technology Institute. Comprehensive portal for humidifier disinfectant damage support: Statistics. Available from: https://www.healthrelief.or.kr/home/content/stats01/view.do.

9. Lee S, Yoon J, Ock J, Jo E-K, Ryu H, Yang W, et al. Individual Exposure Characteristics to Humidifier Disinfectant according to Exposure Classification Groups-Focusing on 4-1 and 4-2 Applicants. Journal of Environmental Health Sciences 2019;45:370-380.

10. Shin H-Y, Kim J, Lee S, Park MS, Park S, Huh S. Cause-of-death statistics in 2018 in the Republic of Korea. Journal of the Korean Medical Association/Taehan Uisa Hyophoe Chi 2020;63

11. Nam M-W, Park S-Y, Suh B-S, Ham S-Y, Son K-H, Kim H, et al. Problems with diagnostic criteria for humidifier disinfectant lung injury (HDLI): two cases of radiologically improved HDLI. Annals of occupational and environmental medicine 2020;32

12. Leem JH, Lee J-H. Humidifier disinfectant-associated specific diseases should be called together as “humidifier disinfectant syndrome”. Environmental health and toxicology 2017;32:e2017017.
